# Effects of Diclofenac, L-NAME, L-Arginine, and Pentadecapeptide BPC 157 on Gastrointestinal, Liver, and Brain Lesions, Failed Anastomosis, and Intestinal Adaptation Deterioration in 24 Hour-Short-Bowel Rats

**DOI:** 10.1371/journal.pone.0162590

**Published:** 2016-09-14

**Authors:** Nermin Lojo, Zarko Rasic, Anita Zenko Sever, Danijela Kolenc, Darko Vukusic, Domagoj Drmic, Ivan Zoricic, Marko Sever, Sven Seiwerth, Predrag Sikiric

**Affiliations:** 1 Department of Pharmacology, School of Medicine, University of Zagreb, POB 916, Salata 11, 10000 Zagreb, Croatia; 2 Department of Pathology, School of Medicine, University of Zagreb, Salata 9, 10000 Zagreb, Croatia; National Cancer Institute, UNITED STATES

## Abstract

Stable gastric pentadecapeptide BPC 157 was previously used to ameliorate wound healing following major surgery and counteract diclofenac toxicity. To resolve the increasing early risks following major massive small bowel resectioning surgery, diclofenac combined with nitric oxide (NO) system blockade was used, suggesting therapy with BPC 157 and the nitric oxide synthase (NOS substrate) L-arginine, is efficacious. Immediately after anastomosis creation, short-bowel rats were untreated or administered intraperitoneal diclofenac (12 mg/kg), BPC 157 (10 μg/kg or 10 ng/kg), L-NG-nitroarginine methyl ester (L-NAME, 5 mg/kg), L-arginine (100 mg/kg) alone or combined, and assessed 24 h later. Short-bowel rats exhibited poor anastomosis healing, failed intestine adaptation, and gastrointestinal, liver, and brain lesions, which worsened with diclofenac. This was gradually ameliorated by immediate therapy with BPC 157 and L-arginine. Contrastingly, NOS-blocker L-NAME induced further aggravation and lesions gradually worsened. Specifically, rats with surgery alone exhibited mild stomach/duodenum lesions, considerable liver lesions, and severe cerebral/hippocampal lesions while those also administered diclofenac showed widespread severe lesions in the gastrointestinal tract, liver, cerebellar nuclear/Purkinje cells, and cerebrum/hippocampus. Rats subjected to surgery, diclofenac, and L-NAME exhibited the mentioned lesions, worsening anastomosis, and macro/microscopical necrosis. Thus, rats subjected to surgery alone showed evidence of deterioration. Furtheremore, rats subjected to surgery and administered diclofenac showed worse symptoms, than the rats subjected to surgery alone did. Rats subjected to surgery combined with diclofenac and L-NAME showed the worst deterioration. Rats subjected to surgery exhibited habitual adaptation of the remaining small intestine, which was markedly reversed in rats subjected to surgery and diclofenac, and those with surgery, diclofenac, and L-NAME. BPC 157 completely ameliorated symptoms in massive intestinal resection-, massive intestinal resection plus diclofenac-, and massive intestinal resection plus diclofenac plus L-NAME-treated short bowel rats that presented with cyclooxygenase (COX)-NO-system inhibition. L-arginine ameliorated only L-NAME-induced aggravation of symptoms in rats subjected to massive intestinal resection and administered diclofenac plus L-NAME.

## Introduction

Massive small bowel resection is imperative in the management of several pathological conditions and is associated with high operative mortality [1] and extended post-operative analgesic use [2].

Therefore, we focused on the early disturbances that occur 24 h after massive intestinal resection (only 20% of the small intestine was retained in rats) including poor anastomosis and failed adaptation of the retained intestine, as well as concomitant occurrence of gastrointestinal, liver, and brain lesions that have been poorly described to date. Then, we focused on massive resectioning with diclofenac administration and the possible noxious stimuli that could further aggravate these particularly dysfunctions including a nitric oxide synthase (NOS)-blocker and L-NG-nitroarginine methyl ester (L-NAME) administered alone or together, after short-bowel formation. In particular, we considered the possible extended analgesic use [2] and also focused on potential adjunct therapy including stable gastric pentadecapeptide BPC 157 and the NOS-substrate L-arginine [3,4], and their underlying mechanisms. The cyclooxygenase (COX) and nitric oxide (NO) systems are not considered to play critical roles after massive small intestine resection, particularly in the initial period.

Counteracting nonsteroidal anti-inflammatory drug (NSAID) toxicity [4–9] particularly, diclofenac-induced [4,7], the stable gastric pentadecapeptide BPC 157 might rescue short-bowel rats in the long-term after massive small intestine resection and improve intestinal anastomosis healing. In addition, it may interact with the NO-system in different animal models and species [3,10]. Consistent with its initial clinical application [11,12] BPC 157 was originally used as an anti-ulcer peptide and thought to be a novel mediator of Robert’s cytoprotection. It is stable in human gastric juice and was designated as a novel mediator of cytoprotection [13]. It has been administered in inflammatory bowel disease [11,12], a multiple sclerosis trial [14], and was recently reviewed [3,4,10–14]. Finally, BPC 157 shows beneficial effects on the NO-system and, thereby, affects gastrointestinal lesions [15,16], fistulas [17–20], blood pressure [15,16], and arrhythmias [16,21].

Furthermore, BPC 157 induced wound healing including in blood vessels [9,10,22–29] and stimulation of the *early growth response 1* (*egr-1*) gene and its co-repressor nerve growth factor 1-A binding protein-2 (naB2) [26]. These factors are also responsible for cytokine and growth factor generation and, therefore, early extracellular matrix (collagen) blood vessel formation [26]. On the other hand, studies have correlated the beneficial effects of BPC 157 with the activation of a cellular focal adhesion kinase (FAK)-paxillin signal pathway and subsequently demonstrated that it dose- and time-dependently increased the expression of the growth hormone receptor, Janus kinase 2 (JAK-2) downstream in the associated signal pathway [27–29]. Furthermore, we hypothesized that the massive small bowel resection early state and concomitant gastrointestinal, liver, and brain lesions may be a particular COX-NO-system inhibition phenomenon. Therefore, in this study we investigated whether BPC 157 and L-arginine [3,4] could act as a specific therapeutic combination and alleviate the initial symptoms as well as modulate the NSAID post-surgery administration and NO-system involvement.

## Materials and Methods

### Animals

Study protocols were conducted in male laboratory rats; strain Albino Wistar, body weight between 250–280 g for males, 14 weeks old, in house bred—animal facility Pharmacology- School of Medicine, Zagreb Croatia. Animal facility registered by Directorate of Veterinary; Reg. No: HR-POK-007. Laboratory rats were acclimated for 5 days and randomly assigned to their respective treatment group. Laboratory animals were housed in PC cages in conventional laboratory conditions at the temperature of 20–24°C, relative humidity of 40-70% and noise level 60 DCB. Each cage was identified following dates: number of study, group, dose, number and sex of each animal. Fluorescent lighting provided illumination 12 hours per day. Standard GLP diet and fresh water was provided *ad libitum*. Animal care was in compliance with SOPs of Pharmacology Animal facility; the European convention for the protection of vertebrate animals used for experimental and other scientific purposes (ETS 123).

Ethical principles of the study ensured compliance with European Directive 010/63/E, the Law on Amendments to Animal Protection Act (Official Gazette 37/13, the Animal Protection Act (official Gazette 135/06), Ordinance on the protection of animals used for scientific purposes (Official Gazette 55/13), FELASA recommendations and recommendations of the Ethics Committee School of Medicine, University of Zagreb. All experiments received specific approval from the Local Ethics Committee at the School of Medicine (University of Zagreb, Zagreb, Croatia). We randomly assigned at least 10 rats per experimental group and period for all experiments.

### Surgery

Using previously described methods [1], a massive small bowel resection was performed (80% removed) under deep anesthesia. The peritoneal cavity was penetrated through a 4-cm midline incision and a single-layer jejunoileal anastomosis performed using 7–0 polypropylene (Prolene, Ethicon, Hamburg, Germany) continuous sutures, and the abdominal incision was closed using 3–0 silk sutures.

### Drugs

The medication, without carrier or peptidase inhibitor, included BPC 157 (a partial sequence of the human gastric juice protein BPC, freely soluble in saline and water at pH 7.0). It was prepared as a peptide with 99% purity (high-performance liquid chromatography, HPLC; main impurity, 1-des-Gly peptide, GEPPPGKPADDAGLV, MW 1419; Diagen, Ljubljana, Slovenia). Diclofenac (Voltaren, Pliva) was prepared as described before [1,4–8,30]. Diclofenac (12 mg/kg) was administered once intraperitoneally, immediately after anastomosis creation. BPC 157 (10 μg/kg and 10 ng/kg), L-NAME (5 mg/kg), and L-arginine (100 mg/kg) were administered per se or combined with each other while controls were administered equivolume saline (5 mL/kg) intraperitoneally.

Diazepam (5mg/kg b.w. i.p.) and sodium thiopental (5mg/kg b.w. i.p.) were used for anaesthesia, while solely sodium thiopental (20mg/kg b.w. i.p) was used for euthanasia.

Due all animals were sacrificed 24 hours after anaesthesia and surgical procedure, we monitored them through those 24 hours and did not administrate any additional drugs to provide additional analgesia.

### Evaluations

The evaluations (24 h after massive small bowel resection) included a macroscopic assessment, histological examination, and biochemical testing performed as described previously [1,3–8,14,30] in the following sections. The evaluations included all animals underwent experiments due there were not any unexpected deaths of animals due to either anaesthesia/medication or surgical procedure.

### Anastomosis assessment

The macroscopy and microscopy assessments were carried out as previously described [1,14,30]. Briefly, the gross anastomosis presentation was scored as 1, total dehiscence; 2, partial dehiscence; 3, edema; and 4, normal anastomosis. The adhesion presentation was scored from 0–7: 0, no adhesion; 1, thin adhesions covering less than half of the anastomosis; 2, prominent adhesions on more than half of the anastomosis; 3, exaggerated adhesions with whole anastomosis; 4, the mesenterial part of the small bowel was also involved; 5, neighboring small intestine loop also involved; 6, numerous neighboring small intestine loops involved; and 7, neighboring loops, the stomach, and were liver ‘packed’. Intestinal passage obstruction [30] was scored from 0–3 according to the loop diameter ratios close to the anastomosis. In other words, an oral loop diameter of 2 cm/aboral loop diameter of 2 cm = 1 denoted a normal passage (score 0); ratios 1–1.33, mild obstruction (score 1); 1.33–1.66, moderate obstruction (score 2); and < 1.66, severe obstruction (score 3).

For microscopy assessment [1,14,30], the tissue specimens were immediately fixed in buffered formalin (pH 7.4) for 24 h, dehydrated, and then embedded in paraffin wax. The samples were stained with hematoxylin and eosin (H&E, for evaluation of epithelization, necrosis, edema, granulation tissue, and inflammatory cells) and examined in a blinded fashion. For the morphometrical analysis, special software programs SFORM and ISSA (Vamstec, Zagreb, Croatia) were used. Five high power fields (HPFs) were randomly selected for the analysis. We determined the edema scores as none (0); gap not exceeding one-third or one thickness of muscle layer area (1); gap exceeding one thickness but not two thicknesses of muscle layer area (2); and gap of two thicknesses of the muscle layer area (3). Necrosis and granulation tissue formation were scored as follows: none (0); present on less than 20% of the anastomosis area (1); present on 20–60% of the anastomosis area (2); and present on more than 60% of the anastomosis area (3). Morphometrical analyses were used to determine the total number of inflammatory cells, reticulin, and collagen present (expressed as a percentage of the total area).

To assess adaptation of the small intestine, we used previously described methods [1] to measure a region 3-cm proximal to 3-cm distal to the anastomosis, villus height (from villus base to villus tip), crypt depth (from crypt base to villus base), and muscle thickness (inner, circular, and outer, longitudinal muscular layer) in blinded fashion. We used an objective mounted micrometer (200×magnification) and optical microscope at 10× and 100× magnification. Data for the mucosal height (villus height and depth) and muscle thickness (inner, circular, and outer, longitudinal, muscular layer) are averages of eight measurements for each animal. In accordance with previous findings villus height, crypt depth, inner circular muscle thickness, and outer longitudinal muscular layer measurements of 307.4 ± 13, 145.2 ± 14.7, 22.6 ± 5.8, and 12.5 ± 4.6 μm, respectively in the ileum and 414.6 ± 14.4, 165.2 ± 12.3, 27.1 ±6.7, 15.5 ± 4.0 μm, respectively in the jejunum were considered normal [1].

### Gastrointestinal lesion assay

The gastric, duodenal, and small and large intestinal lesions were determined as described previously [1,3–8,14,30]. Injury severity was assessed immediately after euthanasia. The sum of the longest lesion diameters was assessed as described previously [1,3–8,14,30], and tissues were processed for routine microscopic analysis as described previously [1,3–8,14,30].

### Liver lesions

The liver tissue samples were immediately placed in 10% neutral buffered formalin for 24 h and subsequently embedded in paraffin. The H&E stained sections were analyzed in three high-power fields. The number of nuclei was counted and their diameters measured using the ISSA program (Vamstec, Zagreb, Croatia) and the number of binucleated cells was also counted. Microvesicular steatosis was scored from 1–3: 1, less than 20% of hepatocytes showed microvesicular steatosis; 2, 20–60% of hepatocytes showed microvesicular steatosis; and 3, over 60% of hepatocytes showed microvesicular steatosis. Parenchymal necrosis, eosinophilic cytoplasm, pyknotic nuclei, and conspicuous nucleoli were scored semiquantitatively as follows: 0, showed no changes; 1, minimum; 2, moderate; and 3, maximum changes [5–7].

### Bilirubin and enzyme activity

To determine the serum levels of aspartate transaminase (AST), alanine transaminase (ALT, IU/L), and total bilirubin (μmol/L), blood samples were collected immediately after euthanasia and centrifuged for 15 min at 3000 rpm. All tests were performed using an Olympus AU2700 analyzer with original test reagents (Olympus Diagnostica, Lismeehan, Ireland) [5–7].

### Brain lesions

As previously described [5–7] the whole brain was fixed in 10% neutral buffered formalin for 2 days, grossly inspected, and then consecutive 5-μm coronal sections were cut. Brain slabs were dehydrated in graded ethanol, embedded in paraffin, and the blocks were cut into 5-μm-thick sections, which were subsequently deparaffinized in xylene, rehydrated in graded ethanol, and H&E stained. The intensity and distribution of brain lesions (swollen or damaged hypoxic neurons) and edema were described and evaluated semi-quantitatively with 0 indicated no changes. The lesions were then scored on a 0–3 scale, edema (1, weak diffuse, perifocal or both; 2, moderate; 3, intense and generalized) and 0–4, ballooned or red neurons (1, 2, 3, and 4 were < 5, 5–30, 30–50, and >50% red neurons, respectively. It is noteworthy to mention that neurons are particularly vulnerable to damage from hypoxia, which causes distinctive histological ischemic changes including pronounced cytoplasmic eosinophilia (red neurons), collapse of cytoplasm with accentuated pericellular spaces, and pyknotic nuclei with indistinct nucleoli. In addition, ischemia produced ultrastructural cellular necrotic changes with breaks in the nuclear and cell membranes and flocculent densities in the mitochondria.

### Statistical analysis

Nonparametric Kruskal-Wallis and Mann–Whitney U-tests were used for the statistical analysis. The number of rats presenting with epithelization or newly formed muscle was compared using Fisher’s two-tailed exact probability test. Values of P < 0.05 were considered statistically significant.

## Results

Post-surgical examination (24 h) revealed the beneficial effects of BPC 157(Tables 1 and 2, Figs 1, 2, 3, 4, 5, 6, 7, 8, 9, 10 and 11) on the poor anastomosis associated with the massive small intestine resectioning, and initial adaptation attempts of the remaining intestine (Table 1 and Figs 1 and 2). The lesions presented rapidly in the upper gastrointestinal tract (Fig 3), liver (Figs 4 and 5), and brain (Table 2 and Fig 6).

**Table 1:** Microscopy and gross anastomosis assessment (min/med/max)(days) in short bowel-rats and in short bowel-rats that received diclofenac (12 mg/kg intraperitoneally).

Microscopy and gross anastomosis assessment											
Parameters assessed	Massive small bowel resection		Massive small bowel resection								
			Diclofenac								
	saline	BPC157 μg	saline	BPC157 μg	BPC 157 ng	L-NAME	L-arg	L-NAME+L-arg	L-NAME+BPC 157μg	L-arg +BPC 157 μg	L-NAME+L-arg+BPC 157μg
Edemascored 0–3	3/3/3	1/1/1*	3/3/3	1/1/1*	1/1/1*	3/3/3	2/2/2*	3/3/3	1/1/2*	1/1/1*	1/1/2*
Granulationscored 0–3	0/0/0	0/0/0	0/0/0	0/0/0	0/0/0	0/0/0	0/0/0	0/0/0	0/0/0	0/0/0	0/0/0
Necrosis scored 0–3	2/2/2	1/1/1*	3/3/3	2/2/2	2/2/2	3/3/3*	2/3/3	3/3/3*	2/2/2	2/2/2	2/2/2
Number of inflammatory cells	150/169/185	120/135/154*	170/189/ 205	135/148/164*	145/159/172*	190/219/ 225*	160/180/ 195	180/189/ 195*	143/153/169*	133/140/163*	130/158/170*
Intestinal obstruction scored 0–3	1/1/1	0/0/0*	1/1/1	0/0/0*	0/0/0*	1/1/2	1/1/1	1/1/1	0/0/0*	0/0/0*	0/0/0*
Adhesions scored 0–7	1/1/1	0/0/0*	1/1/1	0/0/0*	0/0/0*	1/1/2	1/1/1	1/1/1	0/0/0*	0/0/0*	0/0/0*

BPC 157 (10 μg/kg, 10 ng/kg), L-NAME (5 mg/kg), L-arginine (100 mg/kg) were applied alone and/or combined while controls received an equivolume of saline (5 ml/kg) intraperitoneally.

*P<0.05, at least, vs. control

**Table 2:** Brain lesions (scored 0–4, or 0–3, Min/Med/Max) in short bowel-rats and in short bowel-rats that received diclofenac (12 mg/kg intraperitoneally).

Brain lesions											
Brain edemascored 0–3	Massive small bowel resection		Massive small bowel resection								
			Diclofenac								
Balonized or red neuronsscored 0–4	sali-ne	BPC157 μg	sali-ne	BPC157 μg	BPC 157 ng	L-NAME	L-arg	L-NAME+L-arg	L-NAME+BPC 157 μg	L-arg+BPC 157 μg	L-NAME+L-arg+BPC 157μg
Edema	3/3/3	0/0/0*	3/3/3	1/1/1*	1/1/1*	3/3/3	2/2/2*	3/3/3	1/1/2*	1/1/1*	1/1/2*
Cerebral	3/3/3	1/1/1*	3/3/3	1/1/1*	1/1/1*	*4/4/4*	2/2/2*	3/3/3	1/1/2*	1/1/1*	1/1/2*
Cerebellar nn	2/2/2	1/1/1*	4/4/4	1/1/1*	1/1/1*	*4/4/4*	2/3/3*	4/4/4	2/2/2*	2/2/2*	2/2/2*
Purkinje cells	1/1/1	0/0/0*	2/2/2	1/1/1*	1/1/1*	*3/3/3*	2/2/2*	2/2/2	1/1/1*	1/1/1*	1/1/1*
Hippocam-pus	3/3/3	1/1/1*	3/3/3	1/1/1*	1/1/1*	*4/4/4*	2/2/2*	3/3/3	1/1/1*	1/1/1*	1/1/1*

BPC 157 (10 μg/kg, 10 ng/kg), L-NAME (5 mg/kg), L-arginine (100 mg/kg) were applied alone and/or combined while controls received an equivolume of saline (5 ml/kg) intraperitoneally.

*P<0.05, at least, vs. control.

**Fig 1:**
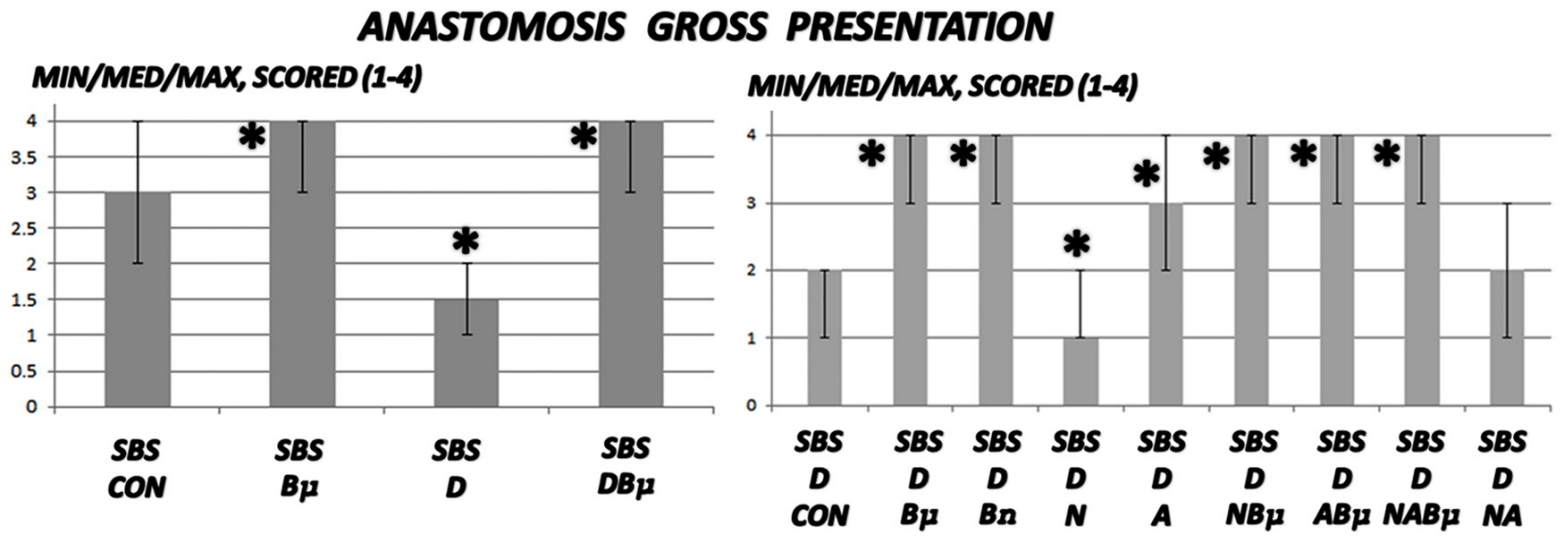
Gross anastomosis assessment. Gross anastomosis was scored 1–4 (min/med/max) in untreated 24 h-short bowel-rats (SBS) and those administered diclofenac (D, 12 mg/kg intraperitoneally). BPC 157 (Bμ and Bn, 10 μg/kg and 10 ng/kg, respectively), L-NAME (N, 5 mg/kg), and L-arginine (A, 100 mg/kg) were administered alone or combined while controls received equivolume saline (5 mL/kg) intraperitoneally. *P<0.05, at least, vs. control.

**Fig 2:**
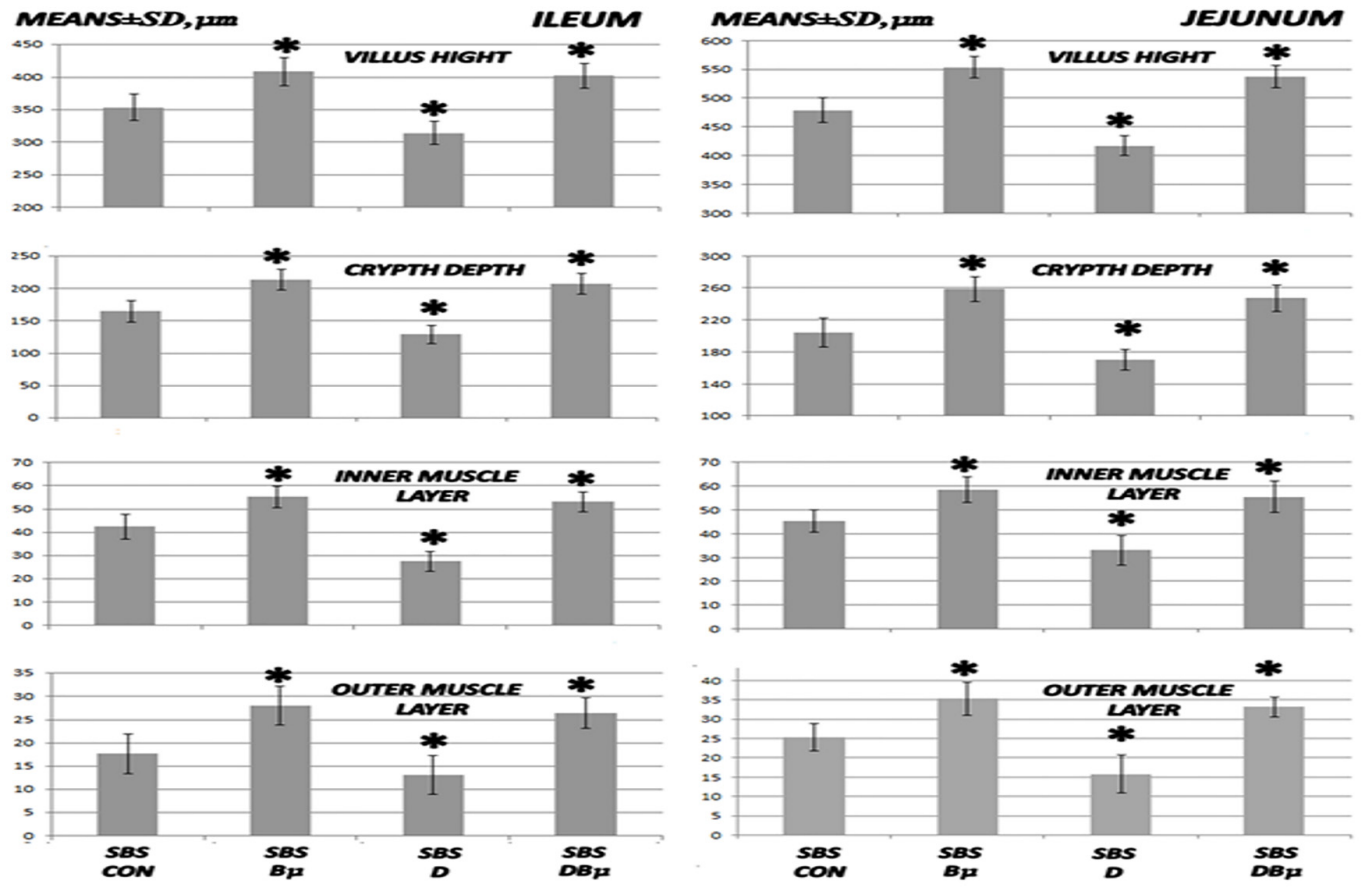
Microscopy of villus height, crypt depth, and inner and outer muscle layer adaptation I. Microscopical assessment of villus height, crypt depth, and inner and outer muscle layer (means ± SD, μm) adaptation in 24 h-short bowel-rats (SBS) administered diclofenac (D, 12 mg/kg intraperitoneally), BPC 157 (Bμ, 10 μg/kg) alone or combined, and controls administered equivolume saline (5 mL/kg) intraperitoneally. *P<0.05, at least, vs. control.

**Fig 3:**
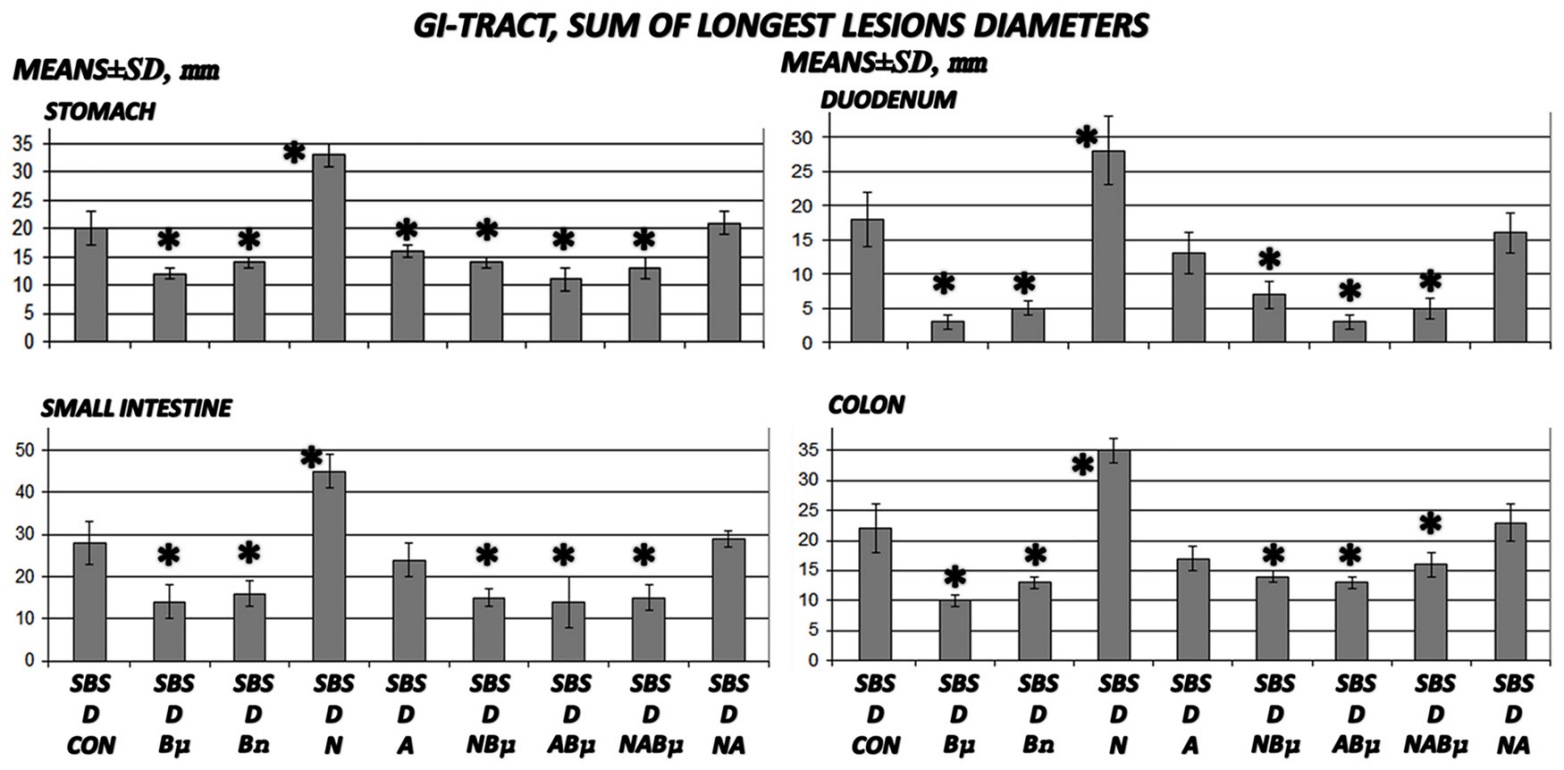
Gross gastrointestinal lesion assessment in 24 h-short bowel-rats administered diclofenac. The sum of the longest diameters of gross gastrointestinal (means ± SD) in 24 h-short bowel-rats (SBS) administered diclofenac (D, 12 mg/kg intraperitoneally). BPC 157 (Bμ and Bn, 10 μg/kg and 10 ng/kg, respectively), L-NAME (N, 5 mg/kg), and L-arginine (A, 100 mg/kg) were administered alone or combined while controls were administered equivolume saline (5 mL/kg, intraperitoneally. *P<0.05, at least, vs. control.

**Fig 4:**
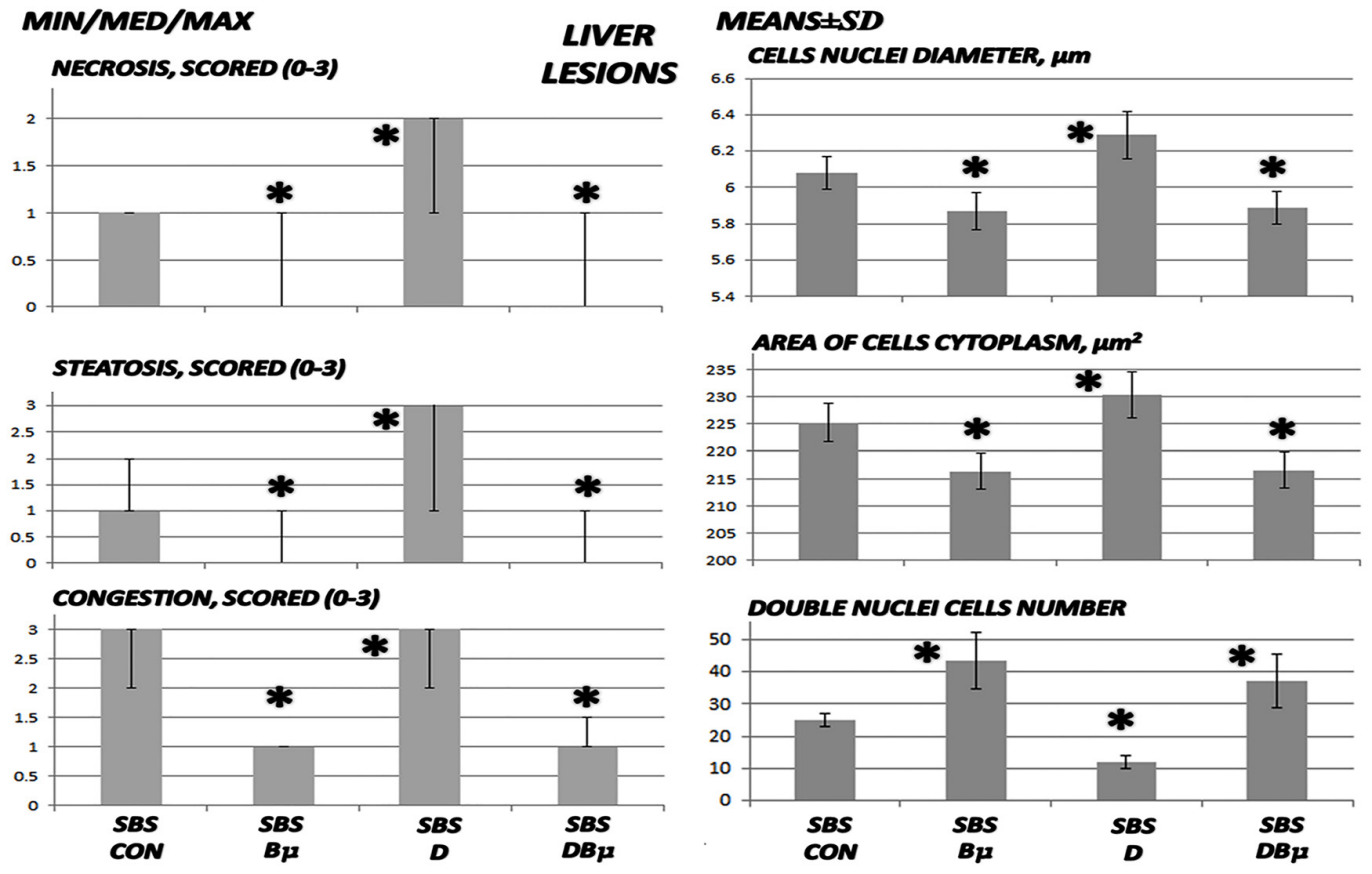
Liver lesion assessment I. Liver lesions including necrosis, steatosis, and congestion were scored as 0–3 (min/med/max), cell nuclei diameters (μm), area of cell cytoplasm (μm^2^), and double nuclei cell number (means ± SD) in untreated 24 h-short bowel-rats (SBS) those administered diclofenac (D, 12 mg/kg intraperitoneally and BPC 157 (Bμ, 10 μg/kg) alone or combined and controls administered equivolume saline (5 mL/kg) intraperitoneally. *P<0.05, at least, vs. control.

**Fig 5:**
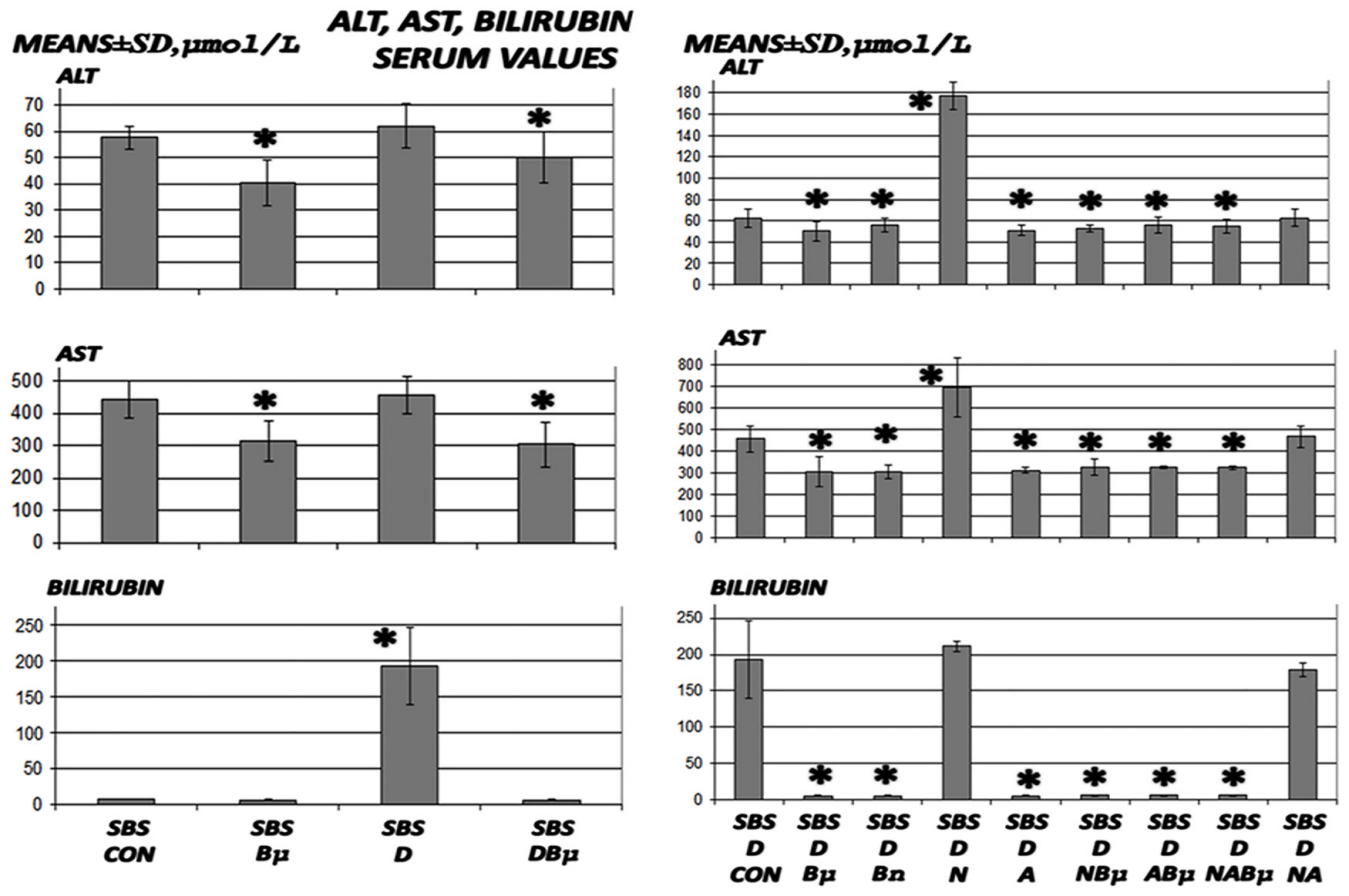
Bilirubin and liver enzymes assessment. Bilirubin and liver enzymes were measured (means ± SD) in untreated 24 h-short bowel-rats (SBS) and those administered diclofenac (D, 12 mg/kg intraperitoneally). BPC 157 (Bμ and Bn, 10 μg/kg and 10 ng/kg, respectively), L-NAME (N, 5 mg/kg), and L-arginine (A, 100 mg/kg) were administered alone or combined and controls were administered equivolume saline (5 mL/kg) intraperitoneally. *P<0.05, at least, vs. control.

**Fig 6:**
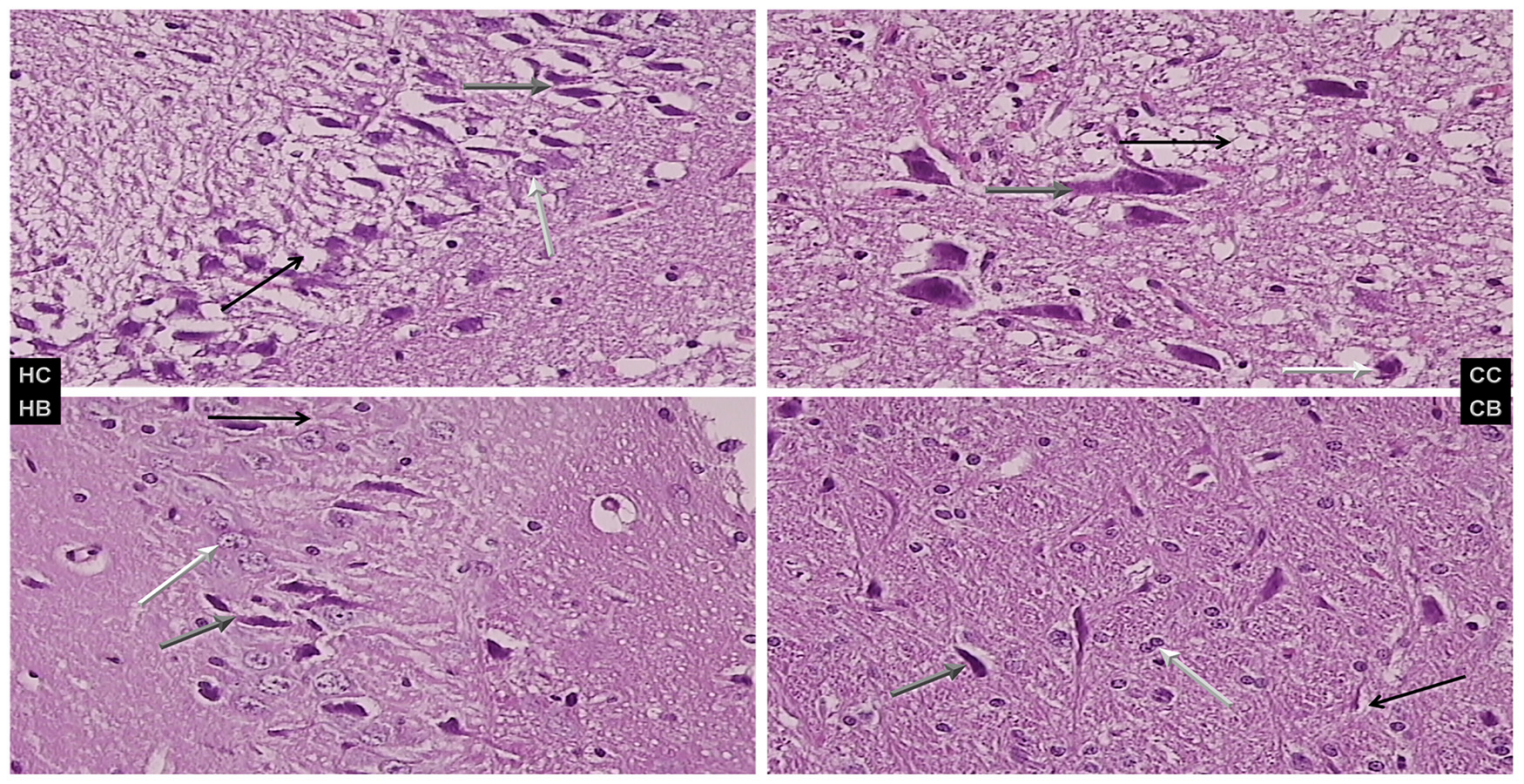
Brain lesion evaluations. Brain lesions were assessed in short bowel rats administered diclofenac (D, 12 mg/kg), saline 5 mL/kg, or BPC 157 (10 μg/kg) intraperitoneally. Characteristic presentation of hippocampal (HC and HB) and cerebellar nuclear (CC and CB) lesions are shown. Left upper panel (control, HC) shows severe edema and neuronal damage (gray arrow indicates ballooned; black, edematous; and white, normal neurons); right upper panel (BPC 157, HB) shows less edema and neuronal damage (gray arrow indicates ballooned; black, edematous; and white normal neurons). Cerebellar nuclei, lower panel (control (HC) shows severe edema and neuronal damage (gray arrow indicates ballooned; black, edematous; and white normal neurons); right lower (BPC 157, HB) shows less edema and neuronal damage (gray arrow indicates ballooned; black, edema; and white, normal neurons), H&E, 40×.

**Fig 7:**
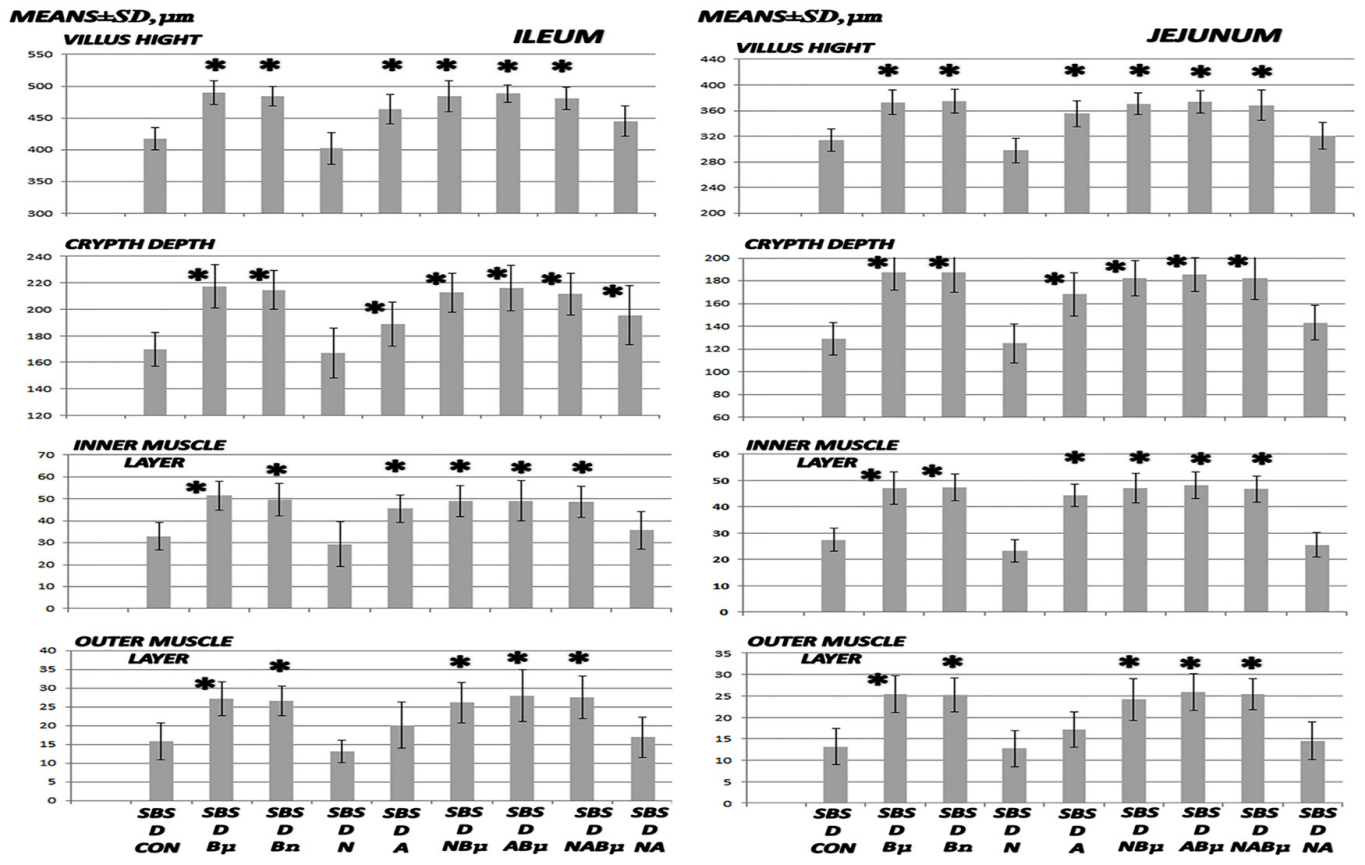
Microscopy of villus height, crypt depth, and inner and outer muscle layer adaptation II. Microscopical assessment of villus height, crypt depth, and inner and outer muscle layer adaptation (means ± SD, μm) in 24 h-short bowel-rats (SBS) treated with diclofenac (D, 12 mg/kg intraperitoneally). BPC 157 (Bμ and Bn, 10 μg/kg and 10 ng/kg, respectively), L-NAME (N, 5 mg/kg), and L-arginine (A, 100 mg/kg) were administered alone or combined while controls were administered equivolume saline (5 mL/kg) intraperitoneally. *P < 0.05 vs. control.

**Fig 8:**
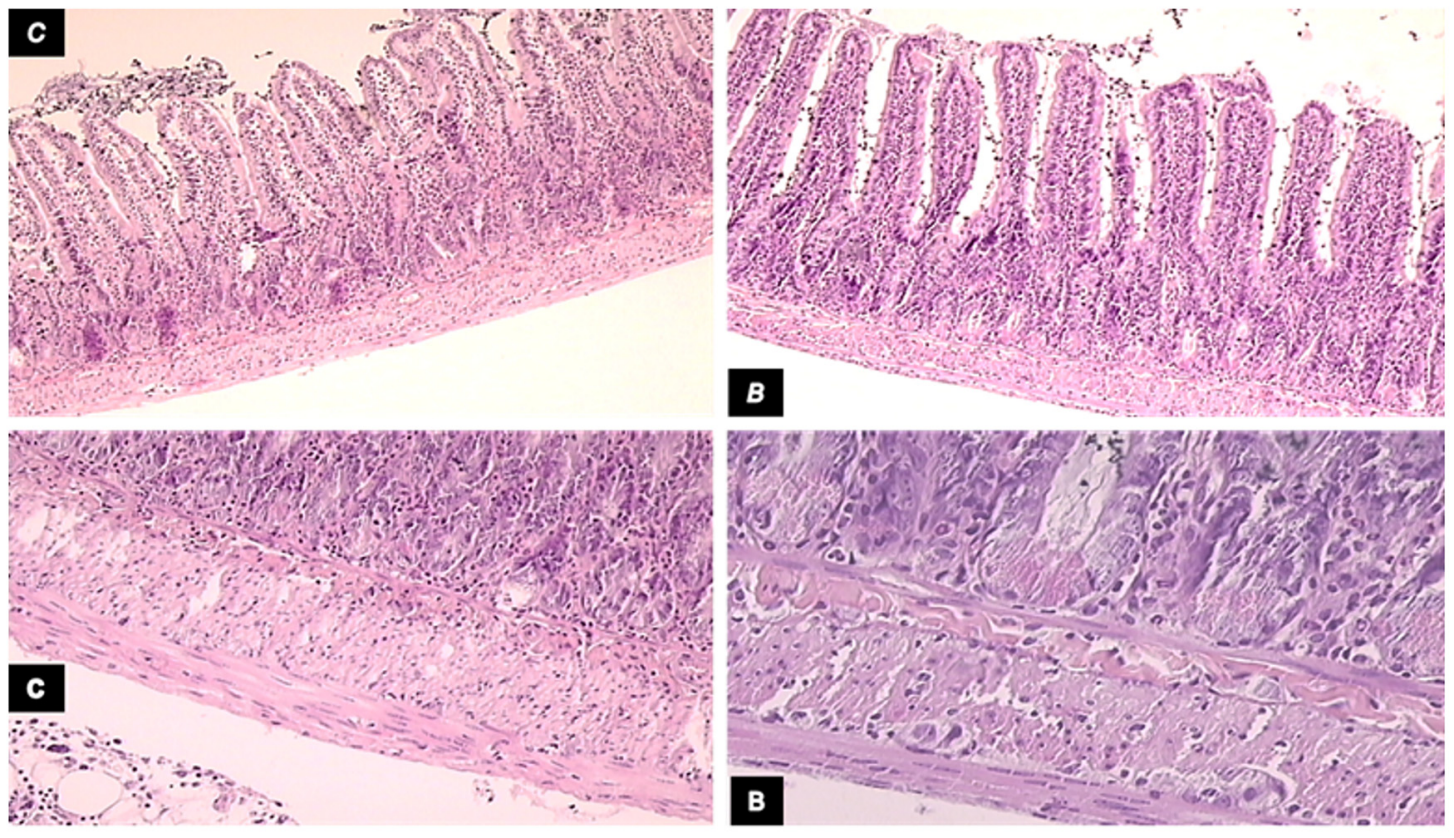
Microscopic presentation of intestinal adaptation in diclofenac-treated animals. Microscopic presentation of intestinal adaptation in diclofenac-treated, controls (C), and BPC 157-treated (B) 24 h-short bowel rats. Upper left, control (C) shows lower mean villus height and shallower crypt depth, H&E, 10×); upper right, BPC 157 (B) shows increased villus height and crypt depth, H&E, 10×; lower panel, control (c) shows muscle thickness was less increased (H&E, 20×; and lower panel, BPC 157 (B) shows muscle thickness was increased more H&E, 20×).

**Fig 9:**
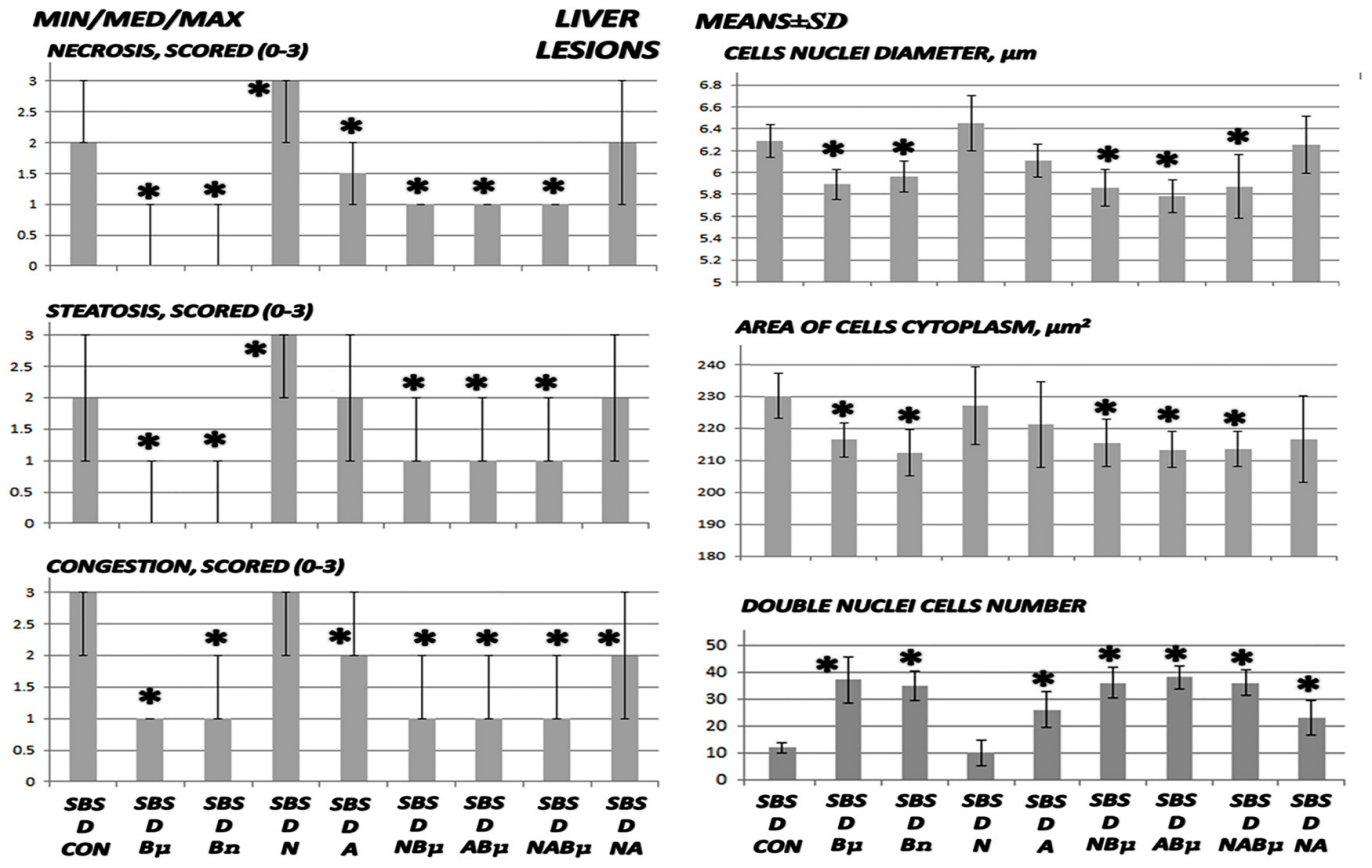
Liver lesion assessment II. Liver lesion assessment revealed necrosis, steatosis, and congestion and was scored as 0–3 (min/med/max), cell nuclei diameters (μm), area of cell cytoplasm (μm^2^), double nuclei cell number (means ± SD) in untreated 24 h-short bowel-rats (SBS) and those administered diclofenac (D, 12 mg/kg intraperitoneally). BPC 157 (Bμ and Bn, 10 μg/kg and 10 ng/kg, respectively), L-NAME (N, 5 mg/kg), and L-arginine (A, 100 mg/kg) were administered alone or combined while controls were administered equivolume saline (5 mL/kg) intraperitoneally. *P < 0.05 vs. control.

**Fig 10:**
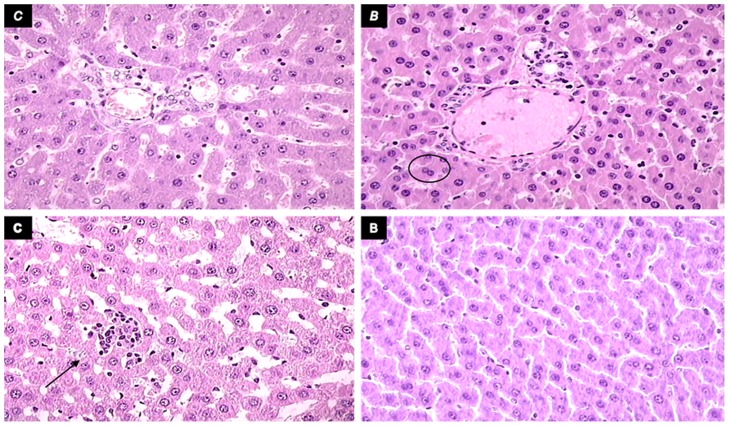
Liver lesions presentation. Liver lesions in short bowel rats administered diclofenac (12 mg/kg), saline 5 mL/kg, or BPC 157 (10 μg/kg) intraperitoneally. Upper left panel, control (c) shows extensive microvesicular steatosis and sinusoidal dilatation, H&E, 40×. Upper right panel, BPC 157 (B) shows less to no steatosis and minor sinusoidal dilatation (circle) and more binucleated hepatocytes (H&E, 40×). Lower panel, control (c) shows occasional focal necrosis (arrow, HE, 40×). Lower panel, BPC 157 (b) shows no necrosis, H&E, 40×.

**Fig 11:**
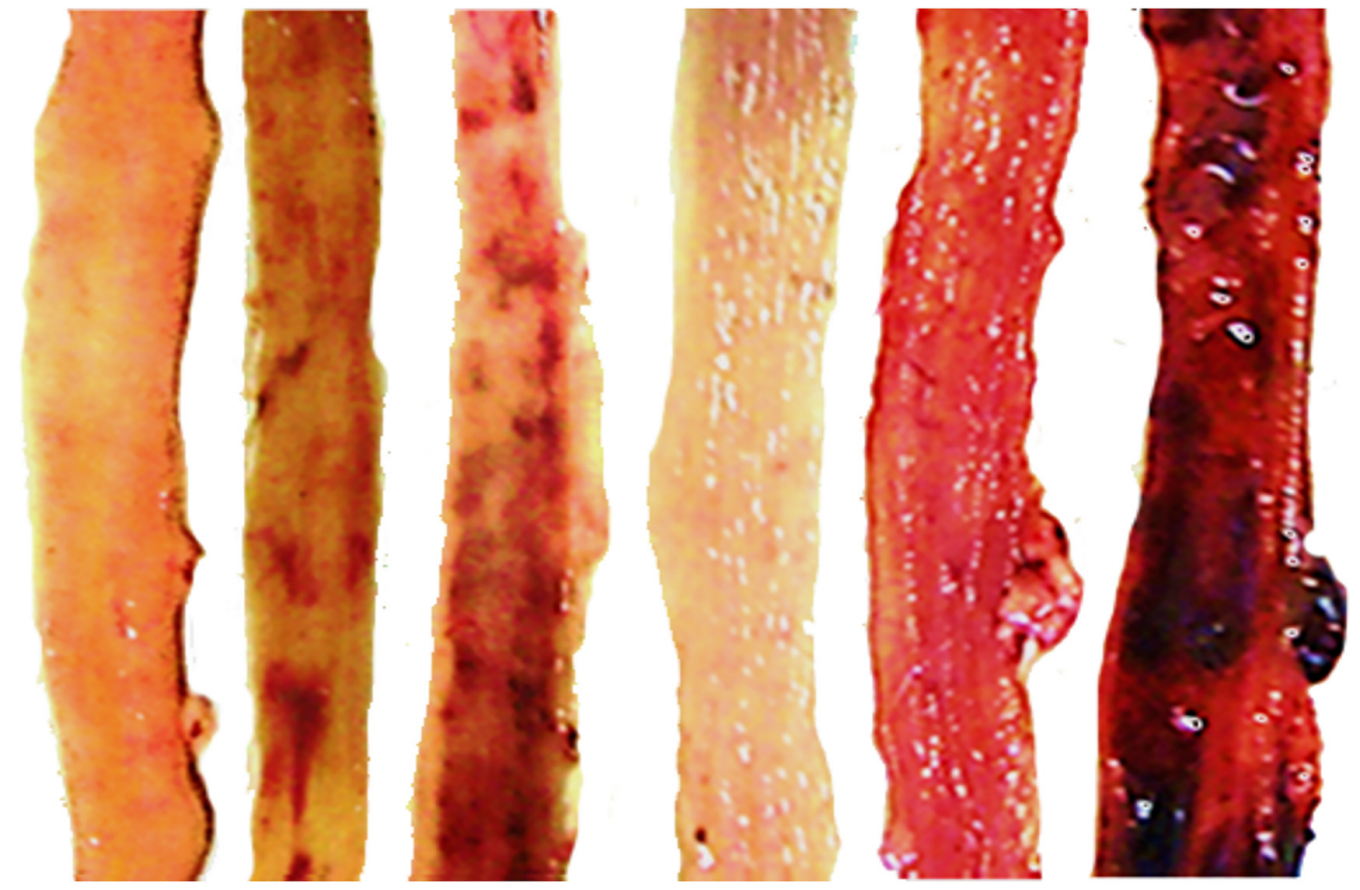
Characteristic gross lesions in 24 h-short bowel-rats treated with diclofenac (small intestine and colon). Characteristic gross lesion presentation in 24 h-short bowel-rats treated with diclofenac in the small intestine (left, +BPC 157, +saline, and +L-NAME) and colon (right, +BPC 157, +saline, and +L-NAME).

Then, assessment of symptom aggravation (Tables 1 and 2, Figs 1, 3, 5, and 7–10) revealed early deterioration following administration of diclofenac, with widespread lesions in the whole gastrointestinal tract. In addition, a higher level of deterioration occurred when diclofenac and L-NAME were combined while this effect was ameliorated by the stablepentadecapeptide BPC 157 and attenuated to a lesser degree by L-arginine.

### Short bowel and anastomosis presentation

As revealed 24 h after massive small intestine resection, anastomosis as well as gross and microscopic presentations corresponded with ongoing adaptation in the remaining intestine. (Table 1 and Figs 1, 2, 7, and 8).

Diclofenac was shown to worsen the poor gross anastomosis presentation (without gross dehiscence), which was complicated by edema, increased inflammatory cells, and necrosis after massive small intestine resectioning. In addition, this effect was even more intense in rats administered diclofenac and L-NAME (Table 1, Fig 1). BPC 157 improved both gross and microscopic presentation of anastomosis after massive small intestine resectioning and completely counteracted the diclofenac-induced exacerbation in rats, as well as additional aggravation in rats administered the combination of diclofenac and L-NAME. Furthermore, L-arginine alone showed a beneficial effect on some assessed parameters. No further beneficial effect was observed when BPC 157 and L-arginine coadministered. Indicatively, while L-arginine reduced the L-NAME-aggravation to the levels of the diclofenac-controls, BPC 157 was more effective, completely reversing the L-NAME-induced damage and anastomosis to normal presentations.

More intense habitual adaptation occurred in all the intestinal layers of both the ileum and jejunum in the remaining small intestine in rats administered BPC 157 (normally, ongoing adaptation is greater in muscle layers than it is in the mucosa, Figs 2 and 8). In contrast, decay appeared in rats administered diclofenac after the massive small intestine resectioning and this limited ongoing adaptation was reversed. Following BPC 157 co-administration, the negative diclofenac-reversal returned to the original BPC 157-mediated improved values in all intestinal layers of both the ileum and jejunum of the remaining small intestines. Furthermore, considering the impaired adaptation following the massive small intestine resectioning in rats administered diclofenac (Fig 7), L-arginine alone showed no effect but it lowered the L-NAME-aggravation to levels that were similar to the diclofenac-control values. Indicatively, unlike the diclofenac plus L-arginine-, diclofenac plus L-NAME-, and diclofenac plus L-NAME plus L-arginine-treated rats, which showed no improvements, BPC 157 improved the diclofenac-induced adaptation as well as the additional impairment caused by L-NAME in all of the wall layers. No further beneficial effects were observed when BPC 157 and L-arginine were co-administered.

### Gastrointestinal lesions

Short-bowel rats habitually presented with small lesions in the stomach and duodenum (2.5 ± 1.0 and 2.0 ± 1.0 mm, respectively), which were completely attenuated in rats administered BPC 157. It is noteworthy that administration of diclofenac led to severe widespread lesions in the whole gastrointestinal tract, stomach, duodenum, small intestine, and colon, which were exacerbated following coadministration of diclofenac and L-NAME (Figs 3 and 11).

Interestingly, in massive small intestine resectioned and diclofenac-treated rats, L-arginine alone showed no effects but appeared to antagonize the L-NAME-induced exacerbation (diclofenac plus L-NAME plus L-arginine-treated rats exhibited lesions similar to those of diclofenac-treated rats). BPC 157, in all groups and all combinations consistently exhibited a beneficial effect and in particular, appeared to nullify the effect of L-NAME, with lesions markedly below diclofenac control values. No potentiation of the beneficial effect was observed following coadministration of L-arginine and BPC 157.

### Liver lesions, bilirubin levels, and enzyme activity

Liver lesions, bilirubin levels, and enzyme activity are presented in Figs 4, 5, 9, and Fig 10. Short-bowel rats habitually presented with liver lesions (Figs 4 and 10) as well as increased serum values of ALT, AST, and particularly bilirubin (Fig 5), which were all markedly exacerbated in rats administered diclofenac, and even more so in rats coadministered diclofenac and L-NAME (Figs 5 and 9).

In general, BPC 157 improved symptoms in short-bowel rats by ameliorating the habitual liver lesions, as well as completely reversing the diclofenac-induced exacerbation of liver lesions and increased serum values of ALT, AST, and particularly bilirubin in all treated groups (BPC 157, 10 μg/kg and 10 ng/kg; L-NAME + BPC 157; L-arginine + BPC 157; and L-NAME + L-arginine + BPC 157).

Furthermore, in diclofenac-treated rats, L-arginine alone showed a beneficial effect. No further beneficial potentiation was observed when BPC 157 and L-arginine were coadministered. Indicating that, while L-arginine reduced the L-NAME-induced aggravation to the values of the diclofenac-controls, BPC 157 was more effective and completely reversed the L-NAME-induced damage.

These effects can gradually exaggerate the only the mild stomach and duodenal mucosal lesions that occur in massive small intestine resectioning. However, there is still evidence of mild liver lesions as well as the already existing severe cerebral/hippocampal lesions. As the symptoms were exacerbated with widespread severe lesions in the whole gastrointestinal tract, the liver lesions were exacerbated as well. In addition, the aggravation of the cerebellar nuclea/Purkinje cells lesions and the cerebral/hippocampus lesions were further aggravated in rats. All these lesions (in particular, cerebral/Purkinje cells/hippocampus lesions) were even more intense in rats coadministered L-NAME and diclofenac after massive small intestine resectioning.

### Brain

The brain lesion evaluations are presented in Table 2 and Fig 6. Moderate and severe edema (more often in the white matter) with red neurons (more often in the gray matter) without any inflammatory reaction were also reduced by BPC 157 administration. Of note is the fact that this beneficial effect was concomitant with an improvement in all intestinal layer post-resection adaptation and lesions in the gastrointestinal tract and liver.

After a massive small bowel resection, Purkinje cells commonly appear to be least affected, cerebellar nuclei are more affected, and cerebral cortical and hippocampal neurons are most affected.

The combination of massive small bowel resectioning and diclofenac, enhanced their individual toxicities as well and, therefore, the cerebellar nuclei, cerebral cortex, Purkinje cells, and hippocampal neurons were all severely affected. These effects were further aggravated in rats coadministered diclofenac and L-NAME (in particular, cerebral/Purkinje cells/hippocampal lesions).

Of note, in diclofenac-treated rats, in all groups and combinations, BPC 157 consistently exhibited a beneficial effect and in particular, might have nullified the effect of L-NAME with lesions markedly below diclofenac-control values. Furthermore, in diclofenac-rats, L-arginine alone showed a beneficial effect. Again, no further beneficial potentiation was observed when BPC 157 and L-arginine were coadministered. However, L-arginine reduced the L-NAME-induced aggravation to the values of the controls while BPC 157 was more effective than L-arginine and completely reversed L-NAME-induced damage.

Therefore, taking the brain lesions as the end result, the reduction in brain lesions supports the evidence that BPC 157 counteracted the deterioration from the onset. In addition, other beneficial effects of BPC 157 administration were observed in different NSAID-induced brain injuries [5–7].

## Discussion

We have attempted to illustrate likely risks associated with major surgery and extended possible analgesic use in patients [2] including poor initial healing, lesions occurring in other organs during the most critical early period, COX-blockade, and additional NOS-blockade, in a rat model permitting acute evaluation within 24 hours. Thereby, during the period immediately following surgery, short-bowel rats presented with gastrointestinal tract, liver, and brain lesions, as well as failed anastomosis healing, intestinal adaptation aggravation by COX-blockade (diclofenac), and further exacerbation by additional NOS-blockade (diclofenac plus L-NAME). In addition, we also sought to determine possible benefit of the use of the stable gastric pentadecapeptide BPC 157 and NOS-substrate L-arginine [3,4], which have not been previously shown.

Importantly, BPC 157 fully counteracted the lesions in rats subjected to massive resectioning combined with the effects of diclofenac and L-NAME. In addition, BPC 157 apparently reduced all pernicious failure induced by the COX- and NOS-system in this early post-surgery syndrome (diclofenac-, L-NAME-, diclofenac plus L-NAME-rats). This was in addition to the previously observed beneficial effect shown in long-term short-bowel studies [1], as well as its counteraction of NSAID-induced toxicity [4] and NOS-blockade [3].

The NO system may be specifically involved [3] in the L-arginine-induced the inhibition of L-NAME-induced exacerbation of the symptoms. The more severe diclofenac plus L-NAME-induced lesions were abrogated by L-arginine (to the diclofenac control level, diclofenac plus L-NAME plus L-arginine-treated rats) (but not below the control values, which was achieved with BPC 157 (diclofenac plus L-NAME plus BPC 157-treated rats)) rather than the regular diclofenac lesions (mediated by COX inhibition and effects of diclofenac) which remained unopposed.

On the other hand, considering the similar mechanisms involved in modulating COX and NOS inhibition [30–33], in the early short-bowel rats, diclofenac likely increased COX and NOS inhibition, suggesting a further NOS-system failure [32,33]. This is an undesirable consequence that occurred in conjunction with, and probably as a contributor to, the additional widespread lesions in rats coadministered L-NAME and diclofenac, after massive small intestine resectioning. In addition, the inhibition of high levels of NO by NOS inhibitors has been found to decrease the production of prostaglandins in *in vitro* and *ex vivo* models [31]. This explains the previous evidence that BPC 157 might counteract the toxicity of various NSAIDs [4–9], and ultimately counteract L-NAME-effects better than L-arginine does [3].

Finally, the differential beneficial effects of BPC 157 (COX and NOS blockade), L-arginine (NOS-blockade), and L-arginine-independent BPC 157 effects, might support the proposition that BPC 157 and L-arginine may act via two separate NO system pathways as previously suggested [3].

We have previously noted that the central nervous system is particularly vulnerable after massive bowel resection surgery. This central defenselessness is likely more complex than previously believed. Furthermore, less gastrointestinal lesioning reduces the chances of toxins penetrating the liver and brain [7]. The most severe lesions were noted in the cerebral and hippocampal regions accompanied by fewer mucosal lesions in the stomach and duodenum only, or mild (liver) lesions. These legions were exacerbated following treatment with diclofenac (cerebellar/Purkinje cell) and definitively aggravated with L-NAME treatment. These effects appear to be associated with the subsequent spreading lesions in the entire digestive tract, increased liver lesions severity, and the events involved in the anastomosis. These events included macro/microscopic necrosis, which were exacerbated more in rats administered diclofenac alone or combined with L-NAME. Similarly, the already initiated (but limited) habitual adaptation of the remaining small intestine was markedly attenuated and even reversed in diclofenac-treated rats as well as in diclofenac and L-NAME-treated rats. Only BPC 157 consistently (even with coadministration of diclofenac and L-NAME) rescued the rats from the anastomosis, and vigorous intestine adaptation (villus height, crypt depth, inner muscular layer, and outer muscular layers all vigorously adapted). In addition, the BPC 157-treated rats showed fewer liver and brain lesions than the untreated rats did.

Finally, BPC 157 exhibited benefits specifically related to its molecular healing effects [26–29] including stimulation of both the *egr-1* gene and its repressor, Nab2, which could be part of a feedback mechanism that regulates *egr-1*-mediated gene transcription, cytokine and growth factor generation, and early extracellular matrix (collagen) and blood vessels formation [26–29]. Accordingly, BPC 157 might counteract various gastrointestinal [1,6–8,14,15–20,30,34,35], liver [5–7,36–38], and brain [5–7,14,38,39] damages as previously reviewed [3,4,10–13]. Furthermore, besides opposing the gastrointestinal tract, liver, and brain lesions induced by NSAIDs [4–7] and NOS-blockade [3], there is evidence that BPC 157 antagonizes all stomach, liver, and brain associated lesions (including seizures) induced by an overdose of insulin [38]. In this way, it also consistently opposes the associated gastrointestinal tract, liver, and brain lesion axis that is rapidly provoked after massive small bowel resection.

Therefore, the BPC 157-mediated inhibition of the combined NSAID-toxicity and NOS-blockade [3,4], likely counteracted the exaggerated risks associated with the surgery, the NO system likely involved in the gastrointestinal [37] and liver damage, hepatic encephalopathy [38], and the diclofenac-induced intestinal injury, in particular, mediated by the NO system pathways [39]. The validation of hypothesis [3,4] would emphasize the beneficial effects of BPC 157 and encourage its use in post-massive small bowel resection syndrome from the very early stage, which would simultaneously revert the imbalance in both the dysfunctional prostaglandin and NO systems [3,4].

## References

[1] Sever M, Klicek R, Radic B, Brcic L, Zoricic I, Drmic D, et al Gastric pentadecapeptide BPC 157 and short bowel syndrome in rats. Dig Dis Sci. 2009; 54: 2070–2083. 10.1007/s10620-008-0598-y 19093208

[2] Klein M, Krarup PM, Kongsbak MB, Agren MS, Gögenur I, Jorgensen LN, et al Effect of postoperative diclofenac on anastomotic healing, skin wounds and subcutaneous collagen accumulation: a randomized, blinded, placebo-controlled, experimental study. Eur Surg Res. 2012; 48: 73–78. 10.1159/000336208 22343935

[3] Sikiric P, Seiwerth S, Rucman R, Turkovic B, Rokotov DS, Brcic L, et al Stable gastric pentadecapeptide BPC 157-NO-system relation. Curr Pharm Des. 2014; 20: 1126–1135. 23755725 10.2174/13816128113190990411

[4] Sikiric P, Seiwerth S, Rucman R, Turkovic B, Rokotov DS, Brcic L, et al Toxicity by NSAIDs. Counteraction by stable gastric pentadecapeptide BPC 157. Curr Pharm Des. 2013; 19: 76–83. 22950504 10.2174/13816128130111

[5] Ilic S, Drmic D, Zarkovic K, Kolenc D, Coric M, Brcic L, et al High hepatotoxic dose of paracetamol produces generalized convulsions and brain damage in rats. A counteraction with the stable gastric pentadecapeptide BPC 157 (PL 14736). J Physiol Pharmacol. 2010; 61: 241–250. 20436226

[6] Ilic S, Drmic D, Zarkovic K, Kolenc D, Brcic L, Radic B, et al Ibuprofen hepatic encephalopathy, hepatomegaly, gastric lesion and gastric pentadecapeptide BPC 157 in rats. Eur J Pharmacol. 2011; 667: 322–329. 10.1016/j.ejphar.2011.05.038 21645505

[7] Ilic S, Drmic D, Franjic S, Kolenc D, Coric M, Brcic L, et al Pentadecapeptide BPC 157 and its effects on a NSAID toxicity model: diclofenac-induced gastrointestinal, liver, and encephalopathy lesions. Life Sci. 2011; 88(11–12): 535–542. 10.1016/j.lfs.2011.01.015 21295044

[8] Sikiric P, Seiwerth S, Grabarevic Z, Rucman R, Petek M, Jagic V, et al Pentadecapeptide BPC 157 positively affects both non-steroidal anti-inflammatory agent-induced gastrointestinal lesions and adjuvant arthritis in rats. J Physiol Paris. 1997; 91: 113–122. 9403784 10.1016/s0928-4257(97)89474-0

[9] Stupnisek M, Franjic S, Drmic D, Hrelec M, Kolenc D, Radic B, et al Pentadecapeptide BPC 157 reduces bleeding time and thrombocytopenia after amputation in rats treated with heparin, warfarin or aspirin. Thromb Res. 2012; 129: 652–659. 10.1016/j.thromres.2011.07.035 21840572

[10] Seiwerth S, Brcic L, Vuletic LB, Kolenc D, Aralica G, Misic M, et al BPC 157 and blood vessels. Curr Pharm Des. 2014; 20: 1121–1125. 23782145 10.2174/13816128113199990421

[11] Sikiric P, Seiwerth S, Rucman R, Turkovic B, Rokotov DS, Brcic L, et al Focus on ulcerative colitis: stable gastric pentadecapeptide BPC 157. Curr Med Chem. 2012; 19: 126–132. 22300085 10.2174/092986712803414015

[12] Sikiric P, Seiwerth S, Rucman R, Turkovic B, Rokotov DS, Brcic L, et al Stable gastric pentadecapeptide BPC 157: novel therapy in gastrointestinal tract. Curr Pharm Des. 2011; 17: 1612–1632. 21548867 10.2174/138161211796196954

[13] Sikiric P, Seiwerth S, Brcic L, Sever M, Klicek R, Radic B, et al Revised Robert's cytoprotection and adaptive cytoprotection and stable gastric pentadecapeptide BPC 157. Possible significance and implications for novel mediator. Curr Pharm Des. 2010; 16: 1224–1234. 20166993 10.2174/138161210790945977

[14] Klicek R, Kolenc D, Suran J, Drmic D, Brcic L, Aralica G, et al Stable gastric pentadecapeptide BPC 157 heals cysteamine-colitis and colon-colon anastomosis and counteracts cuprizone brain injuries and motor disability. J Physiol Pharmacol. 2013; 64: 597–612. 24304574

[15] Sikirić P, Seiwerth S, Grabarević Z, Rucman R, Petek M, Jagić V, et al The influence of a novel pentadecapeptide, BPC 157, on N(G)-nitro-L-arginine methylester and L-arginine effects on stomach mucosa integrity and blood pressure. Eur J Pharmacol. 1997; 332: 23–33. 9298922 10.1016/s0014-2999(97)01033-9

[16] Barisic I, Balenovic D, Klicek R, Radic B, Nikitovic B, Drmic D, et al Mortal hyperkalemia disturbances in rats are NO-system related. The life saving effect of pentadecapeptide BPC 157. Regul Pept. 2013; 181: 50–66. 10.1016/j.regpep.2012.12.007 23327997

[17] Cesarec V, Becejac T, Misic M, Djakovic Z, Olujic D, Drmic D, et al Pentadecapeptide BPC 157 and the esophagocutaneous fistula healing therapy. Eur J Pharmacol. 2013; 701: 203–212. 10.1016/j.ejphar.2012.11.055 23220707

[18] Klicek R, Sever M, Radic B, Drmic D, Kocman I, Zoricic I, et al Pentadecapeptide BPC 157, in clinical trials as a therapy for inflammatory bowel disease (PL14736), is effective in the healing of colocutaneous fistulas in rats: role of the nitric oxide-system. J Pharmacol Sci. 2008; 108: 7–17. 18818478 10.1254/jphs.fp0072161

[19] Grgic T, Grgic D, Drmic D, Sever AZ, Petrovic I, Sucic M, et al Stable gastric pentadecapeptide BPC 157 heals rat colovesical fistula. Eur J Pharmacol. 2016; 780: 1–7. 10.1016/j.ejphar.2016.02.038 26875638

[20] Baric M, Sever AZ, Vuletic LB, Rasic Z, Sever M, Drmic D, et al Stable gastric pentadecapeptide BPC 157 heals rectovaginal fistula in rats. Life Sci. 2016; 148:63–70. 10.1016/j.lfs.2016.02.029 26872976

[21] Balenovic D, Bencic ML, Udovicic M, Simonji K, Hanzevacki JS, Barisic I, et al Inhibition of methyldigoxin-induced arrhythmias by pentadecapeptide BPC 157: a relation with NO-system. Regul Pept. 2009; 156: 83–89. 10.1016/j.regpep.2009.05.008 19465062

[22] Hrelec M, Klicek R, Brcic L, Brcic I, Cvjetko I, Seiwerth S, et al Abdominal aorta anastomosis in rats and stable gastric pentadecapeptide BPC 157, prophylaxis and therapy. J Physiol Pharmacol. 2009; 60: 161–165. 20388960

[23] Bilic M, Bumber Z, Blagaic AB, Batelja L, Seiwerth S, Sikiric P. The stable gastric pentadecapeptide BPC 157, given locally, improves CO2 laser healing in mice. Burns. 2005; 31: 310–315. 15774286 10.1016/j.burns.2004.10.013

[24] Sikiric P, Seiwerth S, Mise S, Staresinic M, Bedekovic V, Zarkovic N, et al Corticosteroid-impairment of healing and gastric pentadecapeptide BPC-157 creams in burned mice. Burns. 2003; 29: 323–334. 12781609 10.1016/s0305-4179(03)00004-4

[25] Mikus D, Sikiric P, Seiwerth S, Petricevic A, Aralica G, Druzijancic N, et al Pentadecapeptide BPC 157 cream improves burn-wound healing and attenuates burn-gastric lesions in mice. Burns. 2001; 27: 817–827. 11718984 10.1016/s0305-4179(01)00055-9

[26] Tkalcević VI, Cuzić S, Brajsa K, Mildner B, Bokulić A, Situm K, et al Enhancement by PL 14736 of granulation and collagen organization in healing wounds and the potential role of egr-1 expression. Eur J Pharmacol. 2007; 570: 212–221. 17628536 10.1016/j.ejphar.2007.05.072

[27] Chang CH, Tsai WC, Lin MS, Hsu YH, Pang JH. The promoting effect of pentadecapeptide BPC 157 on tendon healing involves tendon outgrowth, cell survival, and cell migration. J Appl Physiol. (1985) 2011; 110: 774–780. 21030672 10.1152/japplphysiol.00945.2010

[28] Chang CH, Tsai WC, Hsu YH, Pang JH. Pentadecapeptide BPC 157 Enhances the Growth Hormone Receptor Expression in Tendon Fibroblasts. Molecules. 2014; 19: 19066–19077. 10.3390/molecules191119066 25415472 PMC6271067

[29] Huang T, Zhang K, Sun L, Xue X, Zhang C, Shu Z, et al Body protective compound-157 enhances alkali-burn wound healing in vivo and promotes proliferation, migration, and angiogenesis in vitro. Drug Des Devel Ther. 2015; 9: 2485–2499. 10.2147/DDDT.S82030 25995620 PMC4425239

[30] Vuksic T, Zoricic I, Brcic L, Sever M, Klicek R, Radic B, et al Stable gastric pentadecapeptide BPC 157 in trials for inflammatory bowel disease (PL-10, PLD-116, PL14736, Pliva, Croatia) heals ileoileal anastomosis in the rat. Surg Today. 2007; 37: 768–777. 17713731 10.1007/s00595-006-3498-9

[31] Salvemini D, Manning PT, Zweifel BS, Seibert K, Connor J, Currie MG, et al Dual inhibition of nitric oxide and prostaglandin production contributes to the antiinflammatory properties of nitric oxide synthase inhibitors. J Clin Invest. 1995; 96: 301–308. 7542281 10.1172/JCI118035 PMC185201

[32] Trettin A, Böhmer A, Suchy MT, Probst I, Staerk U, Stichtenoth DO, et al Effects of paracetamol on NOS, COX, and CYP activity and on oxidative stress in healthy male subjects, rat hepatocytes, and recombinant NOS. Oxid Med Cell Longev. 2014; 2014: 212576 10.1155/2014/212576 24799980 PMC3988730

[33] Murase T, Tian Y, Fang XY, Verbalis JG. Synergistic effects of nitric oxide and prostaglandins on renal escape from vasopressin-induced antidiuresis. Am J Physiol Regul Integr Comp Physiol. 2003; 284: R354–362. 12388460 10.1152/ajpregu.00065.2002

[34] Sikiric P, Seiwerth S, Grabarevic Z, Balen I, Aralica G, Gjurasin M, et al Cysteamine-colon and cysteamine-duodenum lesions in rats. Attenuation by gastric pentadecapeptide BPC 157, cimetidine, ranitidine, atropine, omeprazole, sulphasalazine and methylprednisolone. J Physiol Paris. 2001; 95: 261–270. 11595448 10.1016/s0928-4257(01)00036-5

[35] Bilic I, Zoricic I, Anic T, Separovic J, Stancic-Rokotov D, Mikus D, et al Haloperidol-stomach lesions attenuation by pentadecapeptide BPC 157, omeprazole, bromocriptine, but not atropine, lansoprazole, pantoprazole, ranitidine, cimetidine and misoprostol in mice. Life Sci. 2001; 68: 1905–1912. 11292068 10.1016/s0024-3205(00)01025-0

[36] Prkacin I, Separovic J, Aralicia G, Perovic D, Gjurasin M, Lovric-Bencic M, et al Portal hypertension and liver lesions in chronically alcohol drinking rats prevented and reversed by stable gastric pentadecapeptide BPC 157 (PL-10, PLD-116), and propranolol, but not ranitidine. J Physiol Paris. 2001; 95: 315–324. 11595456 10.1016/s0928-4257(01)00044-4

[37] Sikiric P, Seiwerth S, Grabarevic Z, Rucman R, Petek M, Rotkvic I, et al Hepatoprotective effect of BPC 157, a 15-amino acid peptide, on liver lesions induced by either restraint stress or bile duct and hepatic artery ligation or CCl4 administration. A comparative study with dopamine agonists and somatostatin. Life Sci. 1993; 53: PL291–PL296. 7901724 10.1016/0024-3205(93)90589-u

[38] Ilic S, Brcic I, Mester M, Filipovic M, Sever M, Klicek R, et al (2009) Over-dose insulin and stable gastric pentadecapeptide BPC 157. Attenuated gastric ulcers, seizures, brain lesions, hepatomegaly, fatty liver, breakdown of liver glycogen, profound hypoglycemia and calcification in rats. J Physiol Pharmacol. 2009; 60: 107–114. 20388953

[39] Tudor M, Jandric I, Marovic A, Gjurasin M, Perovic D, Radic B, et al (2010) Traumatic brain injury in mice and pentadecapeptide BPC 157 effect. Regul Pept. 2010; 160: 26–32. 10.1016/j.regpep.2009.11.012 19931318

